# Comparison of mothers and grandmothers physical and mental health and functioning within 6 months after child NICU/PICU death

**DOI:** 10.1186/s13052-018-0531-8

**Published:** 2018-08-10

**Authors:** JoAnne M. Youngblut, Dorothy Brooten

**Affiliations:** Nicole Wertheim College of Nursing & Health Sciences, Florida International University, AHC 3, Rm 241, 11200 SW 8th St., Miami, FL 33199 USA

**Keywords:** Bereaved grandmother health, Bereaved mother health, Grandchild death, Child death, PICU death, NICU death

## Abstract

**Background:**

Losing a child is devastating for parents and grandparents. Family and friends generally focus on comforting and supporting the bereaved parents, unintentionally ignoring the bereaved grandparents. Grandmothers and grandfathers often struggle with wanting to help their adult children (deceased child’s parents) without usurping the parents’ responsibilities and decisions regarding the deceased child. Research on mothers’ and grandmothers’ health at about the same time after the same child’s death in the neonatal or pediatric intensive care unit is lacking. The aim of this study was to compare mothers and grandmothers on physical health, mental health, and functioning in the first 1–6 months after the same child’s death in a neonatal or pediatric intensive care unit.

**Methods:**

This cross-sectional secondary analysis compared 32 mothers with 32 grandmothers of the same 32 deceased children (newborn-6 years). Grandmothers were recruited through these 32 mothers. Most grandmothers and mothers were Hispanic (25%, 34%) or Black (44%, 41%), respectively. Mothers and grandmothers separately completed questions about their Physical Health, Mental Health [depression (Beck Depression Inventory), Post-Traumatic Stress Disorder (PTSD, Impact of Events-R), grief (Hogan Grief Reaction Checklist)], and Functioning (social support [MSPSS] and Employment) since the child’s/grandchild’s death. Paired t-tests and Chi Square tests were used to compare grandmothers with mothers of the same deceased infant/child on their private and separate responses to study measures.

**Results:**

Mothers had significantly more acute illnesses than grandmothers. More mothers (63%) than grandmothers (37%) were categorized as clinically depressed. More mothers (69%) than grandmothers (44%) had clinical PTSD. Mothers reported significantly higher levels of despair and detachment than grandmothers. Only 4 mothers and 2 grandmothers were in therapy at the time of interview. Grandmothers and mothers rated their ability to concentrate on their work and their level of social support similarly.

**Conclusions:**

Mothers had more acute illnesses, more severe depression, and a higher level of grief than grandmothers. However, few received therapy despite their high levels of depressive and PTSD symptoms.

## Background

Death of a child or grandchild is devastating for parents and grandparents with effects on their physical and mental health and functioning. About 43,000 children ages 0–19 years die in the US annually [1], most in intensive care units (ICUs) [2]. The fast paced, high technology and unfamiliar ICU environment is stressful for parents who may watch their infant or child, scared and in pain, for days, weeks, or months before the death. Grandparents are stressed; they often cannot see their grandchild in the ICU because of visitation policies. Some do not live in the same state or country, also limiting their ability to see their critically ill grandchild before death.

Following a child’s death, parents have increased rates of physical and mental illnesses based on Denmark’s national data [3–6]. Data on grandparents’ physical and mental health after grandchild death have been reported in only one cross-sectional study [7]. However, it is not known if outcomes for mothers and grandmothers in the same family differ after the NICU/PICU death of the same child. This study’s aim was to compare grandmothers and mothers on physical health, mental health, and functioning from data collected at about the same time after the same child’s or grandchild’s NICU/PICU death.

In a national database of Danish parents 1–5 years after their child’s death, Li and colleagues [3–6] found increased rates of parent cancer, heart disease, diabetes, mental disorders, and death. In a longitudinal study of relatively young US parents (176 mothers, 73 fathers) (76%, ages 18–40) during the first 13 months after an infant’s/child’s NICU/PICU death [8], the total number of mothers’ chronic health conditions (*n* = 183) more than doubled. Mothers reported 89 hospitalizations, 300 separate episodes of acute illnesses, and 76 medication changes to manage their chronic conditions; most (61%, 76%, and 67%, respectively) occurred in the first 6 months after the child’s death [9]. The proportion of mothers with depression, PTSD, or both decreased from 61 to 71% at 1 month to 25–35% at 13 months. Youngblut et al. [8] found that more Hispanic mothers had clinical depression at 6 months and clinical PTSD at all four time points (1, 3, 6, and 13 months post child death) than White mothers.

Few studies on grandparents’ stress-related health outcomes after a grandchild’s death have been reported. In a cross-sectional study of 99 grandmothers (43% Black, 31% White, 25% Hispanic) and 37 grandfathers (19% Hispanic, 24% Black, 57% White) at 1 to 6 months after their grandchild’s death, Youngblut et al. [7] found that grandparents reported medication changes to manage their chronic health conditions (68%), a total of 59 illnesses (28%) and 7 hospitalizations (4.5%), and rated their health as ≤5 (16%) on a 1 “poor” to 10 “excellent” scale after the grandchild’s death. In addition, grandparents had clinical depression (31%), clinical PTSD (35%), or both (20%); 14% had neither. With statistical control for time since death, Black grandparents had more severe symptoms of PTSD than White grandparents [7]. Of the 8 grandparents in therapy, half had clinical depression and/or PTSD. Results for grandmothers and grandfathers separately were not provided. The study’s cross-sectional design did not capture all illnesses, hospitalizations, medication changes, and mental health problems through 6 months post grandchild death because some grandparent interviews occurred earlier than 6 months, likely resulting in underestimation of grandparent morbidity in the first 6 months.

Parents [10–13] and grandparents [14–18] in separate studies have expressed pain, depression, helplessness, sorrow; shock, numbness, disbelief; bitterness, exhaustion, anger at God; and guilt that the child or grandchild died before them or that they did not do enough to protect or save the child or grandchild, blaming themselves for the child’s or grandchild’s death. Studies of predominantly White parents [11, 19, 20] and White grandparents [14, 18, 21] find that both experience flashbacks of the death, feelings of envy toward others with children or grandchildren, sleep disturbances, increased alcohol and drug use, and thoughts of suicide. However, when a child dies, friends and family focus primarily on the parents. Grandparents are often “forgotten grievers” [16], and they experience greater pain when their loss is not acknowledged [17, 22]. Some grandparents question the legitimacy of their grief [23] or feel their grief is secondary to that of the parents [17]. The grandparent’s role in the grieving family often is not clear, increasing their feelings of isolation.

In several qualitative studies [17, 21–25], parents and grandparents characterized their relationship as strained or distant after the child’s/grandchild’s death. Ponzetti [21] found that 56% of parents and 53% of grandparents reported a change in their feelings toward the other. In another study [18], grandmothers and grandfathers did not differ in their feelings toward their adult child (grandchild’s parent) [14]. Stroebe and colleagues [26] found that Dutch parents felt the need to protect the grandparents by staying strong [27], hiding their feelings and often withholding information [25], increasing parents’ grief. Some studies find that parents’ grief decreases over time [26, 28–30]. One study [31] found a significant drop in mothers’ grief from 6 to 13 months post infant/child NICU/PICU death. However, others [32] continued to find parent grief 3 or more years, and some more than 50 years [33, 34], after a child’s death.

Grandparents’ grief includes the pain of thinking about and watching their grieving adult child (grandchild’s parent) [22], fear of saying something that makes their adult child’s pain worse [15], and unable to help or comfort their adult child [15, 18, 24] and fulfill their role as parent to their adult child [23]. In some studies, grandparents reported trying to “hold in,” suppress, and ignore their grief to protect their adult child and their surviving grandchildren [15, 18, 25]. In studies of parent and grandparent grief, fathers and grandfathers tended to be “strong and silent;” mothers [35, 36] and grandmothers [14, 18, 21] preferred talking through their grief. Mothers and grandmothers also reported a greater need to talk about the deceased child/grandchild than fathers and grandfathers [18, 21, 36]; Israeli grandparents had difficulty finding someone to listen to them [23].

Parent and grandparent role functioning after child or grandchild death is often defined by employment. According to the Society for Human Resource Management [37], employer policies generally provide paid bereavement leave for parents after their child’s death, but not for grandparents after a grandchild’s death. Employment can be a distracter, a source of support or a source of additional stress. In one study, 60% of families lost more than 10% of their annual income after a child’s death and 35% of parents quit their jobs [11]. Grandparents reported that resuming their employment helped them to cope. Parents used work and other activities to distract themselves and to limit or interrupt their ruminating about the deceased child [35]. Reilly-Smorawski et al. reported that mothers often delayed returning to work and were unproductive at work [38, 39].

In summary, a child’s death is devastating for parents and grandparents. Studies document the effects of a child’s death on parents’ physical and mental health. However, research on grandparent physical and mental health and functioning after a grandchild’s death is very limited. The few reported studies found that grandparents had primarily negative mental health (depression, PTSD) and a few reported negative physical symptoms (pain, insomnia). Most studies of parents and grandparents post child death are qualitative, cross-sectional and limited by small, primarily White samples where time since death and the child’s or grandchild’s age at death vary widely within the same study. Gaps in knowledge include lack of research comparing mothers and grandmothers in the same family, especially with a sample that is racially/ethnically diverse with a narrow age range of the deceased children who all died in the NICU or PICU, and data collection in the first 6 months after the child’s death. This study adds to this knowledge base by comparing mothers and grandmothers in the same family on physical and mental health, grief, and functioning in the first 6 months after the same young child’s NICU/PICU death. The investigators hypothesized that mothers would report poorer physical and mental health, greater grief, and poorer functioning than grandmothers.

## Methods

### Design

This secondary analysis combines data from two separate studies of physical and mental health and functioning of mothers and grandmothers after the same child’s death. The first (R01-NR009120) was a longitudinal study of 176 bereaved mothers and 73 fathers where data were collected in a 45- to 60-min oral interview in the parent’s home at 1, 3, 6, and 13 months after the NICU/PICU death of their infant/child (newborn through 18 years). The second (S06-GM008205) was a cross-sectional study of 99 bereaved grandmothers and 37 grandfathers where data were collected in a single 30- to 45-min oral telephone interview between 1 and 6 months after the death of their young grandchild (newborn through 6 years). The data for this study were from the mothers and grandmothers in the same family from both studies whose child or grandchild died in a NICU or PICU. All grandmothers in this secondary analysis were identified and recruited through parents in the first study.

### Sample

The sample consisted of 32 mothers and 32 grandmothers of newborns through 6-year-old children who died in the NICU or PICU in one of four referral hospitals. Mothers (> 18 years old) and grandmothers of infants and children admitted to NICU or PICU at least 1 h before their death and who spoke (but not necessarily read) English or Spanish were eligible for the study. Mothers and grandmothers were not eligible if 1) the deceased newborn was from a multiple gestation pregnancy, 2) the deceased infant/child was living in a foster home before PICU admission, 3) the deceased’s injuries involved child abuse, or 4) a parent or sibling died because of the same event (e.g., motor vehicle crash, childbirth). Additional exclusion criteria for grandmothers were: living in an extended care or skilled nursing facility because of diminished physical and/or cognitive capacity or scoring ≤20 on the telephone Mini-Mental Status Examination [40]. None of the grandmothers were excluded due to either condition.

### Procedures

After approval by Florida International University’s Institutional Review Board and the Institutional or Human Subjects Review Boards at the 4 hospitals and Florida Department of Health for both studies, collaborators at each recruitment site identified potentially eligible parents. Introductory letters (Spanish & English) were mailed to these parents at 2–4 weeks post child NICU/PICU death. A research assistant (RA) called the parents, further explained the study, answered their questions, and scheduled a first data collection visit in the parents’ home, where parents signed informed consent forms for their own participation and review of their child’s medical record. At the end of this first data collection visit, the RA briefly explained the grandparent study to the parents and asked whether the deceased child had eligible grandparents. If parents provided the grandparents’ contact information, a similar introductory letter (Spanish & English) was mailed to the grandparents. One week later, the RA and a second research team member together telephoned the grandparents, further explained the study, answered their questions, read the informed consent form to the grandparent, and obtained each grandparent’s oral informed consent to participate. Both the RA and the second team member signed the oral informed consent form as witnesses to the consent procedure and the grandparent’s oral consent. The IRBs waived written informed consent for grandparents. The 32 grandmothers in this secondary analysis were recruited through the deceased’s mother. The RA then conducted the single telephone oral interview or scheduled it for a time between 1 and 6 months post grandchild death. The RA read the questions to both mothers and grandmothers separately.

### Measures

Mothers and grandmothers provided information about their Physical Health, Mental Health (Beck Depression Inventory [41] (BDI-II), Impact of Events-R [42] (IES-R), Hogan Grief Reaction Checklist [32] (HGRC)) and Functioning (Multidimensional Scale of Perceived Social Support (MSPSS) [43] and information on Employment) since the child’s death.

#### Physical health

Mothers and grandmothers rated their health over the previous 2 weeks compared to others their age from 1 (poor) to 10 (excellent), provided the number of times they had been ill since the death and indicated the type of illness. Mothers and grandmothers were asked whether they had any of the 11 listed chronic conditions and to add other chronic conditions not on the list. For each chronic condition, they indicated whether it was diagnosed before or after the child’s/grandchild’s death.

#### Mental health – Depression

The 21-item BDI-II [41] measures depression. Mothers and grandmothers rated each item from 0 to 3 for the previous 2 weeks. Higher total scores indicate more severe depressive symptomatology. Depression severity groups [41] were created from total scores: None (0–13), Mild (14–19), Moderate/Severe (20–63). Coefficient alphas were .91–.93 for mothers and .93 for grandmothers.

#### Mental health – PTSD

The IES-R [42] measures PTSD and has 3 subscales: intrusiveness of thoughts about the deceased (8 items), avoidance of reminders of the deceased (8 items), and hyperarousal (6 items). Mothers and grandmothers rated its 22 items from 0 (*not at all*) to 4 (*extremely*) to indicate how distressing each had been in the past 2 weeks regarding the death. Total summative scores ≥34 indicate presence of PTSD [42]. Coefficient alphas for the total scale and subscales were .79–.91 for mothers and .86–.94 for grandmothers.

#### Mental health – Grief

Grief was defined as the normal trajectory of grief after loss rather than as the pathological “complicated grief” [30]. The HGRC [32] measures symptoms of “normal” grief in 6 subscales – Despair (13 items), Panic Behaviors (14 items), Blame & Anger (7 items), Detachment (8 items), Disorganization (7 items), and Personal Growth (12 items). Mothers and grandmothers rated the 61 items from 1 (*Does not describe me at all*) to 5 (*Describes me very well*) for the past 2 weeks. Correlations of HGRC subscales with subscales of the Grief Experience Inventory and Texas Revised Inventory of Grief support the HGRC’s validity [32]. Internal consistency subscale reliabilities in this study were .80–.93 for mothers and .83–.94 for grandmothers.

#### Functioning – Social support

The Multidimensional Scale of Perceived Social Support (MSPSS) [43] measures support from family, friends, and a significant other over the previous 2 weeks. Mothers and grandmothers rated each of the 12 items on a 7-point scale from 1 (*very strongly disagree*) to 7 (*very strongly agree*). Higher summative scores indicate greater support. Construct validity is supported by a moderate negative correlation (*r* = −.35) between MSPSS and depression scores for subjects reporting high life stress but no correlation (*r* = .02) for those reporting low life stress [43]. Total scale internal consistencies were .91–.92 for mothers and .90 for grandmothers.

#### Functioning – Employment

Mothers and grandmothers were asked whether they were employed before the death and, if employed after the death, when they returned to their jobs or started a new job. On a scale of 1 (never) to 10 (all the time), mothers and grandmothers rated how often they had trouble focusing on work in the previous 2 weeks.

#### Data analysis

Version 24 of the SPSS statistical software was used for the analyses. Differences in categorized depression, PTSD, and presence of at least 1 chronic condition (yes/no) were tested with Chi Square analyses. Paired t-tests were used for all other comparisons of mothers and grandmothers. Pearson correlations were used to test the strength of the relationship between mothers’ and grandmothers’ paired scores. Alpha level for statistical significance was set at *p* = .05. Analyses reported here include mothers and grandmothers of the same deceased child. Each grandmother was paired with the mother of the same deceased child. All mothers and grandmothers were included only once in the dataset. Data from the mother’s interview closest in time post-death to the paired grandmother’s single interview were used, resulting in 32 grandmother–mother pairs of 32 deceased children/grandchildren. Time from child/grandchild death to interview was not significantly different for the grandmother-mother pairs (mean difference = 0.43, SD = 3.32), paired *t* = 0.74, *p* = .47.

## Results

### Sample description

Age ranges were 38–72 for grandmothers and 19–41 for mothers (Table 1). Most grandmothers and mothers were married or partnered, Hispanic or Black, high school graduates with additional education, and employed before the death. More grandmothers than mothers had returned to their jobs at the time of interview. Most mothers and grandmothers did not expect the child to die (56%, 59%). For 8 mothers and 6 grandmothers the deceased was their first and only child or grandchild. Eight (25%) grandmothers had provided some childcare to the deceased grandchild.

**Table 1:** Characteristics of Mothers and Grandmothers

	Mothers (*n* = 32)	Grandmothers (*n* = 32)
Age [M (SD)]	30 (7.4)	52 (9.3)
Time since child/grandchild death (in weeks)	10.9 (5.77)	11.3 (7.09)
Race/ethnicity [n (%)]		
Black	13 (41%)	14 (44%)
White	8 (25%)	10 (31%)
Hispanic	11 (34%)	8 (25%)
Education [n (%)]		
< High school	2 (6%)	2 (6%)
High school graduate	9 (28%)	11 (35%)
> High school	21 (66%)	19 (59%)
Employed? [n (%)]		
Before death	23 (72%)	22 (69%)
At interview	15 (47%)	20 (63%)
Partnered? [n (%)]	25 (78%)	21 (66%)
Family annual income [n (%)]		
< $20,000	6 (19%)	6 (19%)
$20,000–$49,999	7 (22%)	9 (28%)
≥ $50,000	11 (34%)	14 (44%)
No response	8 (25%)	3 (9%)

Most of the deceased children were infants (69%), male (56%), and died in the NICU (59%) after life support was withdrawn or treatment was limited (63%). Causes of death were primarily congenital anomalies, genetic disorders, and prematurity (Table 2).

**Table 2:** Description of Deceased Infants and Children

	NICU (*n* = 19)	PICU (*n* = 13)
Age group [n (%)]		
Infants	18 (95%)	4 (31%)
Preschool	1 (5%)	8 (61%)
School age	0 (0%)	1 (8%)
Sex [n (%)]		
Female	6 (32%)	5 (38%)
Male	13 (68%)	8 (62%)
Mode of death [n (%)]		
Life support withdrawn	4 (21%)	5 (38%)
Treatment limited	7 (37%)	4 (31%)
Brain death	0 (0%)	3 (23%)
Failed CPR	8 (42%)	1 (8%)
Cause of Death [n (%)]		
Prematurity	7 (37%)	0 (0%)
Congenital anomalies/Genetic disorders	6 (32%)	4 (31%)
Respiratory condition	4 (21%)	0 (0%)
Infection	2 (10%)	2 (15%)
Neurologic condition	0 (0%)	3 (23%)
Accidents	0 (0%)	2 (15%)
Cancer	0 (0%)	1 (8%)
Surgical complications	0 (0%)	1 (8%)

### Physical health

Mothers’ and grandmothers’ scores on self-rated health were similar (Table 3). Grandmothers had significantly more chronic conditions before the child’s/grandchild’s death and in total than mothers, but mothers had significantly more acute illnesses after the child’s/grandchild’s death than grandmothers. Although not statistically significant, twice as many grandmothers (*n* = 23, 66%) reported at least 1 chronic health condition than mothers (*n* = 11, 44%), χ^2^ = 2.49, df = 1, *p* = .12.

**Table 3:** Outcomes for Mothers and Grandmothers

	Mothers M (SD) *N* = 32	Grandmothers M (SD) *N* = 32	Paired t value	Correlation^†^
Physical Health				
Self-rated health	7.3 (2.48)	7.8 (2.00)	0.75	−.08
Total number chronic conditions	0.6 (0.84)	1.4 (1.29)	2.91**	−.23
Number chronic conditions before death	0.2 (0.54)	1.2 (1.26)	3.97**	−.21
Number new chronic conditions after death	0.4 (0.66)	0.3 (0.51)	0.81	−.10
Number acute illnesses after the death	0.7 (0.96)	0.3 (0.57)	2.53*	.13
Mental Health				
Depression (BDI) score	20.0 (13.62)	11.7 (10.52)	2.81**	.05
PTSD (IES-R) score	43.6 (19.51)	35.5 (23.17)	1.73	.27
Hyperarousal	10.9 (7.61)	6.6 (6.97)	2.45*	.10
Avoidance	11.8 (8.05)	11.1 (9.06)	0.40	.31
Intrusion	21.0 (7.22)	17.3 (9.54)	1.87	.12
Grief				
Despair	34.9 (14.90)	27.7 (12.22)	2.29*	.19
Detachment	15.4 (7.88)	11.3 (5.79)	2.23*	.13
Panic	29.8 (13.68)	25.5 (9.22)	1.39	−.09
Blame & Anger	13.7 (7.55)	10.6 (4.82)	1.90	−.10
Disorganization	14.2 (5.51)	13.7 (6.73)	0.39	.13
Personal Growth	38.8 (10.67)	42.3 (11.87)	1.33	.14
Functioning				
Employment – Trouble focusing on work	4.6 (2.97)	5.2 (2.72)	0.86	.05
Social Support	66.3 (13.91)	60.5 (11.06)	1.67	−.18

*p < .05 **p < .01 ^†^All *p* values > .05 (NS)

### Mental health

Categorized BDI scores (clinical depression vs no depression) and IES-R scores (clinical PTSD vs no PTSD) indicated that more mothers than grandmothers had clinical depression or PTSD (63% vs 37% BDI, 69% vs 44% PTSD), although not statistically significant, χ^2^ = 1.28, df = 1, *p* = .26 for BDI and χ^2^ = .72, df = 1, *p* = .40 for PTSD (Table 3). Both women in 11 mother-grandmother pairs had PTSD, and both women in 9 pairs had depression. The IES-R severity scores for hyperarousal symptoms (including excessive emotions, relationship problems, disruptions in sleep and concentration) were significantly higher for mothers than grandmothers (Table 3). Only 4 mothers and 2 grandmothers were in therapy at the time of interview. Mothers reported significantly higher levels of despair and detachment than grandmothers, but similar levels of blame & anger, panic, disorganization, and personal growth.

### Functioning

Grandmothers’ and mothers’ scores for ability to concentrate on their work and level of social support were similar at the time of interview (Table 3).

## Discussion

Very few studies have compared mothers’ and grandmothers’ physical health, mental health, and functioning after the same child’s death. Comparing our findings with other studies’ findings is limited by the lack of quantitative data on grandmothers’ health and functioning and almost no research comparing quantitative data for mothers and grandmothers of the same deceased child. In this study, data were collected from mothers and grandmothers in the same family by the same interviewers at about the same time after the same child’s death. The limited reported studies also focus on White samples.

Both mothers and grandmothers experienced negative physical health effects after the child’s/grandchild’s death. Mothers developed more acute illnesses after the child’s/grandchild’s death than grandmothers. Although grandmothers had more chronic health conditions before the death than mothers, grandmothers and mothers developed about the same number of new chronic health conditions, on average, after the death. More grandmothers (66%) than mothers (44%) reported at least one chronic condition at interview after their grandchild’s/child’s death. Perhaps grandmothers’ acute illnesses after the death were attributed to or masked by their greater number of pre-existing chronic health conditions. With larger samples over the first 6 months post infant/child NICU/PICU death, researchers have reported 302 and 59 acute illnesses, 108 and 75 pre-existing chronic health conditions, 108 and 20 newly-diagnosed chronic conditions, 59 and 19 medication changes, and 59 and 7 hospitalizations for 176 mothers and 99 grandmothers, respectively [7–9]. Mothers’ and grandmothers’ self-ratings of their health were about the same, with means toward the healthier end of the scale.

During the first 6 months after child/grandchild death, mothers experienced more severe depressive symptoms than grandmothers. Clinical depression rates were not statistically different between mothers (63%) and grandmothers (37%); however, they were much higher than for US adults of the same ages – 7.7% of adults age 18–39, 8.4% of adults age 40–59, and 8.0% of adults age 60 and older [44].

Mothers had significantly more severe symptoms of the hyperarousal component of PTSD (excessive emotions, relationship problems, disruptions in sleep and concentration) than grandmothers. While more mothers (69%) than grandmothers (44%) had clinical PTSD scores, this difference was not statistically significant. Comparing our PTSD findings with other studies’ outcome variables was not possible due to lack of data from mothers and grandmothers of the same deceased child in the same time frame. As with clinical depression rates, mothers and grandmothers PTSD rates in this study were considerably higher than PTSD rates (3.6%) among US adults in any given year [45].

Mothers had higher scores than grandmothers on 2 of the 6 grief (HGRC [32]) subscales. Mothers experienced significantly more severe symptoms of despair (hopelessness, sadness, missing the child) and detachment (avoidance of tenderness, withdrawal from others) than grandmothers. Severity of grief symptoms regarding blame & anger (bitter, hostile, vengeful), panic (physiologic response), disorganization (difficulty concentrating, learning, remembering), and personal growth (spiritual, existential awareness) were similar for mothers and grandmothers. Detachment may be greater for mothers in the first 6 months as they distance themselves from their mothers (the deceased’s grandmother) [23]. In another study, most parents whose child (0–48 years old) died up to 62 years earlier reported that this detachment was not long-lasting [46]. The similar scores on the other 4 subscales suggest that grandmothers may have a level of grief close to that of mothers of the same deceased child in these areas.

Both grandparents and parents have reported feelings of bitterness, anger, and self-blame; pain, shock, and hearts pounding; and being unable to concentrate or focus and to remember things [11–18, 21, 23]. Scores on personal growth were low (possible range 12–60) in this sample of mothers and grandmothers, perhaps because of the short time between the child’s/grandchild’s death and the time of interview. The first 6 months after a child’s/grandchild’s death is a period of many health challenges for both mothers and grandmothers [7–9].

Employment is often described as a coping or escape mechanism after a child’s or grandchild’s death [23]. Most grandmothers and about half of the mothers had returned to their jobs by the time of interview. In the larger sample [8], 50% of mothers returned to work by 4 weeks post child NICU/PICU death. Grandmother-mother pairs did not differ on their ratings about their ability to work. However, the means for mothers and grandmothers on this employment item were low, around or below the midpoint (5) of the scale. These findings indicate that both mothers and grandmothers had difficulty managing their work with their grief.

Grandmothers and mothers had similar mean scores on their perceived social support. This is somewhat surprising given the results of qualitative studies of mothers and grandmothers (separate studies) [17, 21–25, 36, 47–49]. When a child dies, the focus is on the parents’ grief, mental health, and functioning, and grandparents are often the “forgotten grievers” [16]. It may be easier for parents to find social support because healthcare providers, clergy, and others have spent more time with the parents than the grandparents prior to the child’s/grandchild’s death and are more aware of the parents’ need for support. Parents, especially mothers, may attend support groups to help them cope [50]. Grandmothers reported relying on their spouse, the grandchild’s mother, friends, and other relatives for support and preferred to talk through their grief [14, 18]. Grandmothers may have to be more active in seeking the support they need.

O’Leary et al. [25] suggested that parents feel a “double burden,” especially with pregnancy loss, because of the loss of their infant and also of disappointing or upsetting their parents (the infant’s grandparents). Others have hypothesized that grandparents experience “double pain” – pain for their deceased grandchild and pain for their grieving adult child (the deceased’s parent) [23, 51]. The few significant differences between mothers and grandmothers in this study raise questions about the strength of the effects of parents’ double burden and grandparents’ double pain on their grief and morbidity. Perhaps the effects of double pain on grandparents and double burden on the parents are of similar magnitude. It is also possible that, when the two occur together, one masks the effect of the other. The present study’s findings may also reflect this sample’s racial/ethnic diversity versus the primarily White samples of other studies.

Thoughts about the appropriate timing for contacting parents after an infant’s/child’s death vary across clinicians, researchers, and others. Bereaved parents in a number of studies have noted the reticence of family, friends, and co-workers to talk with them after the death because they don’t know what to say to the parents or they don’t want to upset the parents [36, 47–49]. Grandparents and parents in Australia, Canada, Israel, The Netherlands, and the US report suppressing or ignoring their grief to protect the other [15, 23, 25, 26].

### Limitations

The small number of women (*n* = 64, 32 mother-grandmother pairs) makes it more difficult to achieve statistical significance and limits the generalizability of the study’s findings. Since grandparents were identified through the parents, provision of the grandparent(s)’ name(s) and contact information may have been affected by the parents’ perception of the grandparent’s health and/or reaction to the grandchild’s death. If parents only provided names of grandparents they perceived as healthy and/or dealing well with the grandchild’s death, this study may underestimate grandparent morbidity. Parents who were estranged from the deceased child’s grandparents would be unable or unlikely to provide names and contact information for them. The possible effect of this factor on the study is not clear.

This study may under- or over-estimate the number of acute illnesses for mothers and grandmothers, since these data were collected as self-report. It may have been difficult for these women to accurately report their acute illnesses since the child’s death. Women may report an acute illness that has lasted for months (e.g. colds, headaches) as 1 episode, missing the fine distinctions between the end of one acute illness and the beginning of another. While this may under-represent the number of illnesses, obtaining these data from individual HCPs or urgent care centers was not feasible in this study. The illnesses recorded in a woman’s chart likely would yield a low count because adults often do not seek formal health care for illnesses like colds and headaches.

Women were categorized as having or not having depression and/or PTSD based on their self-reported severity of symptoms. Diagnosis from a mental health practitioner might be more definitive, although they often gauge the severity of symptoms based on the patient’s report of their symptoms. Women who reported high severity of symptoms, especially suicidality, were encouraged to seek help from a mental health practitioner. At a 6-month data collection visit, one woman was actively suicidal and appropriate steps were taken to ensure immediate assessment and treatment from a mental health practitioner.

## Conclusion

Study findings indicate that mothers had more acute illnesses, more severe depression and hyperarousal, and greater despair and detachment than grandmothers, but grandmother-mother pairs had similar levels of blame and anger, panic behaviors, and disorganization after the same child’s death. Despite their need for therapy, few mothers and grandmothers received it. Primary care providers should consider both physical and mental health when seeing a mother or grandmother after a child’s or grandchild’s death. Future research is needed with a larger, racially/ethnically diverse sample of parent-grandparent dyads followed longitudinally to determine health and functioning effects of a child’s NICU/PICU death on families.

## Abbreviations

BDI: Beck depression inventory II
DAS: Dyadic adjustment scale
DOH: Department of Health
HGRC: Hogan grief reaction checklist
ID: Identification
IES-R: Impact of events-R
IRB: Institutional Review Board
M: Mean
MSPSS: Multidimensional scale of perceived social support
NICU: Neonatal intensive care unit
PICU: Pediatric intensive care unit
PTSD: Post-traumatic stress disorder
RA: Research assistant
SD: Standard deviation
US: United States
